# Winter Diseases

**Published:** 1866-12

**Authors:** 


// HALL'S JOURNAL OF HEALTH.

OUR LEGITIMATE SCOPE IS ALMOST BOUNDLESS: FOR WHATEVER BEGETS PLEASURABLE AND HARMLESS FEELINGS, PROMOTES HEALTH; AND WHATEVER INDUCES DISAGREEABLE SENSATIONS, ENGENDERS DISEASE.

*We aim to show how Disease may be avoided, and that it is best, when sickness comes, to take no Medicine without consulting an educated Physician.*

VOL. XIII.] DECEMBER, 1866. [No. 12.

### WINTER DISEASES

ARE coughs, colds and consumptions, with others more specially fatal; among the latter is Pneumonia, called by some Inflammation of the lungs, and by others Lung Fever. It is a disease which attacks the feeble and the strong; the most rugged are taken to the grave by it, in four or five days; others it spares to live and suffer for years; even those who get over it remain feeble for months.

Pneumonia is one of the avoidable diseases, and being of such a dangerous nature, they are wisest who knowing the causes, seek to avoid them habitually. A man sat up writing till a late hour of a Winter's night; he noticed that the fire had gone out, but thought he could finish what he had in hand in a short time, but it required a longer period than he thought for, and when he had finished he found himself chilled through and through; this was followed by an attack of Pneumonia from which he suffered for two years, running into consumption, of which he died.

A young lady remained at a party in the country until two o' clock of a March morning; there was dancing and music and mirth, the rooms were warm, and she left them when the body was overheated, rode home in an open carriage and found herself completely chilled; she had an attack of lung fever, and at the end of two years stated that she had never seen a well day since the night of the party.

A young lady was sitting in a warm room in her father's house, and some friends called in a carriage; rather than put them to the trouble of coming in, as they merely wanted some item of information, and had an infant with them, she went to the gate to speak with them; it was snowing a little, and becoming interested in some recital, she noticed a chilly feeling pass over her. She never saw a well day afterwards.

Many excellent clergymen have had fatal attacks of pneumonia by failing to throw a cloak over their shoulders after the excitement of preaching in a cold room, or out of doors.

Lung fevers are common with music teachers, who, after the excitement of walking to the residence of their pupils are allowed to wait some moments in cold parlors; or after giving a lesson in a warm room, especially in vocal music, go out into a damp, raw wind.

Clergymen and other public speakers have often been prematurely laid aside, by being compelled, after speaking, to ride several miles on horseback against a cold wind.

An attack of pneumonia is often occasioned by getting into a public vehicle after having been excited by walking, and being compelled to sit in the draft of an open window which some selfish, inconsiderate clown had raised for his own comfort, regardless of any consequences to others.

To remain at rest in any position until a feeling of chilliness is induced, is sufficient to bring on an attack of inflammation of the lungs, however vigorous and robust the person may feel.

Sitting still with damp feet; standing on the wet grass; keeping on damp clothes after having been engaged in exercise, are frequent causes of lung fever. One great principle, practicle in its nature and easily understood, underlies all these cases: it is the getting chilled; this is the more easily brought about in proportion to the amount of exercise which has been previously taken to the extent of inducing a warmth of body above what is natural; the easy and universal preventive is, cool off very slowly after all forms of exercise in cold weather.

If a delicate person goes to bed in a warm room of a cold night and the fire goes entirely out, leaving the room thirty, forty or fifty degrees colder when rising than at the hour of retiring, a cold is a very certain result.

